# Decoding Multidimensional Machining Loads: iKIT Wireless Extrasensory Toolholder and Parametric Analysis in Aluminum Cutting

**DOI:** 10.3390/s26134302

**Published:** 2026-07-07

**Authors:** Qian Qiao, Dawei Guo, Chi-Tat Kwok, Lap Mou Tam

**Affiliations:** 1 IDQ Science and Technology (Hengqin, Guangdong) Co., Ltd., Zhuhai 519000, China; qqiao@idq.org.mo; 2 Institute for the Development and Quality, Macao 999078, China; 3 Department of Electromechanical Engineering, University of Macau, Macao 999078, China; fstctk@um.edu.mo

**Keywords:** wireless extrasensory toolholder, milling dynamics, multi-axis force, aluminum alloy

## Abstract

Smart manufacturing requires real-time monitoring of multidimensional forces at the interface between the tool and workpiece in computer numerical control (CNC) machining. In this study, an innovative iKIT wireless extrasensory toolholder is introduced that is capable of high-fidelity, in situ, high-frequency sensing and monitoring of the cutting force, torque, and two-way bending moments. The hardware design of the system is outlined, highlighting a high-bandwidth miniature wireless transmission method and noncontact power supply and energy storage solution suitable for rotating machining environments. To assess the system performance, comprehensive milling tests were performed on aluminum alloy materials, and the relationship between the process parameters and changes in multidimensional mechanical loads was thoroughly examined. The experimental findings demonstrate that the smart toolholder detects precisely how parameter variations affect the loads. Multidimensional mechanical signals (torque and two-way bending moments) show a strong positive correlation with the feed rate and axial depth of cut, confirming the impact of the material removal rate on the system loads. Conversely, these signals are negatively correlated with spindle speed, accurately reflecting the effects of thermal softening and a reduced friction coefficient in aluminum alloys during high-speed cutting. This study not only offers a dependable hardware framework for integrating miniaturized sensors into toolholders, but also delivers accurate data to support digital twin models and adaptive control in machining processes.

## 1. Introduction

The levels of intelligent manufacturing and automation in the manufacturing sector continue to advance [1]. Recently, the transition towards Industry 5.0 has emphasized human-centric perspectives [2], while novel frameworks are continually proposed to achieve green intelligent manufacturing for net-zero emissions [3]. Furthermore, modular and adaptive architectures have been developed to enhance flexible production [4]. The development of new manufacturing systems and implementation of projects focused on intelligent manufacturing, informatization, automation, and smart technologies have become key trends in the evolution of manufacturing technologies. Intelligent manufacturing is a vital direction for industrial progress [5]. Real-time monitoring of computer numerical control (CNC) machining processes has become essential for enhancing machining quality and production efficiency. This is particularly true in the high-speed milling of aluminum alloys, which are often used in the aerospace and automotive industries [6]. Small variations in the cutting torque and spatial bending moments often reveal early signs of tool wear, chatter, and surface quality degradation. Traditional indirect monitoring methods, such as measuring spindle current and machine tool vibrations, cannot accurately capture the complex mechanical conditions at the tool-workpiece interface owing to signal loss and noise interference over long transmission paths. Therefore, directly acquiring high-fidelity, multidimensional, and in situ mechanical load data from the cutting zone is a prerequisite for enabling adaptive control technologies.

Cutting tools serve as critical connections between machine tools and workpieces and play a fundamental role in machining [7,8]. Currently, intelligent cutting tool product lines are effectively replacing traditional cutting tools. These intelligent tools retain the basic cutting functions of conventional tools while monitoring the machining conditions and tool wear by integrating several sensors. In the current digital era, intelligent cutting tools have become essential components that enable intelligent manufacturing and automated production. For rough machining, tool usage is optimized by intelligent toolholders that monitor cutting forces to provide relevant information about machining status and detect trends, indirectly overseeing chip removal and tool life [9,10]. In precision machining, digital monitoring of tool deflections and instantaneous loads is crucial for preventing unexpected workpiece displacement, uneven surfaces, and ultimately higher scrap rates. This capability enables rapid responses to process deviations, thereby extending tool life and ensuring process repeatability.

Most existing smart toolholders are confined to single-axis torque measurements or the monitoring of axial tensile and compressive stresses. However, in practical high-speed end-milling operations, transverse and lateral bending moments frequently serve as the primary contributors to tool deflection and machining inaccuracies. Accurate measurement of cutting forces, spindle torque, and bidirectional bending moments within the limited spatial constraints of a compact toolholder remains a significant technical challenge [11,12]. Furthermore, integrating a multidimensional sensing system into a high-speed rotating, fluid-intensive environment poses substantial mechatronic difficulties [13]. Among these are challenges associated with high-speed wireless data transmission and the provision of a continuous power supply. Traditional battery-powered approaches are hindered by critical drawbacks, including a limited operational lifespan, frequent maintenance requirements, and susceptibility to compromising the dynamic balance of the toolholder during high-speed rotation [14]. Additionally, electromagnetic shielding within enclosed machine tools, coupled with the centrifugal and Doppler effects induced by rapid spindle rotation, often result in severe packet loss and elevated latency in wireless communication protocols such as Bluetooth and Wi-Fi [15,16]. The absence of a reliable noncontact power supply and high-bandwidth miniaturized wireless transmission system significantly constrains the sustained industrial application of tactile toolholders.

To address these limitations, a structural optimization and iterative experimental validation are conducted, culminating in the deployment of a wireless smart toolholder. This system enables real-time, in situ monitoring of axial force (F), torque (M), and biaxial bending moments ($B_{x}$, $B_{y}$). The foundational physical characteristics of this specific toolholder—including static linearity, repeatability, cross-talk decoupling (maintained below 5%), and dynamic stiffness—were strictly validated against a standard Kistler stationary dynamometer in a previous foundational study [17]. Building upon this validated hardware reliability, the current study conducts comprehensive in-depth milling experiments on aluminum alloy specimens. The fundamental objective is to systematically decouple the influences of cutting speed, feed rate, and axial depth of cut on the multidimensional mechanical signatures, thereby moving beyond basic sensor demonstration to establish a quantitative link between macroscopic cutting parameters and microscopic contact mechanics.

## 2. Experimental Method

### 2.1. Basic Information

The wireless smart toolholder, iKIT-BT40-ER25-130-40-H, was developed by IDQ Science and Technology (Hengqin, Guangdong) Co. Ltd. Zhuhai, China and recorded axial force (*F*), biaxial bending moments (*B_x_* and *B_y_*), and torque (*M*). Detailed information regarding the technical and functional specifications of the wireless extrasensory toolholder have been described previously [17]. To ensure data credibility under dynamic cutting conditions, a full-bridge strain gauge configuration is employed to achieve inherent hardware-level temperature compensation, effectively minimizing thermal drift during prolonged machining. Furthermore, built-in digital low-pass anti-aliasing filters were implemented prior to signal transmission to refine the raw measurements. Regarding the critical issue of dynamic balance caused by integrated electronics and batteries, the toolholder was structurally optimized with symmetrical internal cavities and strategically placed counterweights, achieving an allowable unbalance grade of G2.5 at 25,000 rpm according to the ISO 1940-1 standard [18].

**Figure 1:**
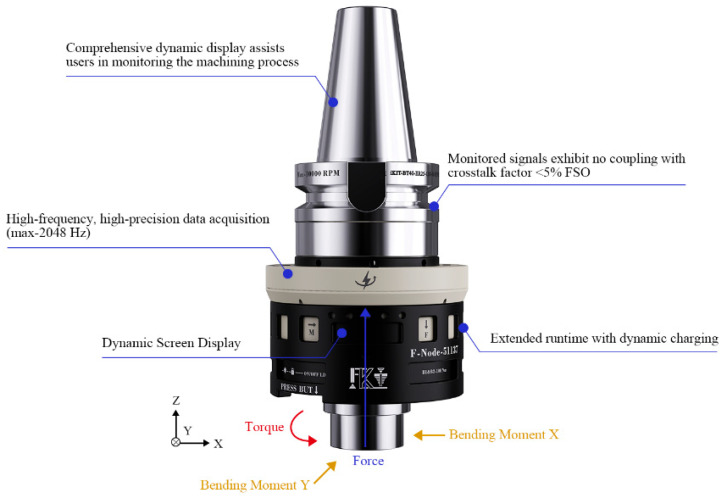
Advantages of the wireless smart toolholder.

The current investigation focuses on clarifying the features of the toolholder and validating its usability through manufacturing trials. The main features of this toolholder are illustrated in Figure 1, and summarized as:

(1) The monitored signal data are presented dynamically and comprehensively in all directions to enable users to oversee the processing;
(2) The latency is maintained within the range of 10–20 ms, which is significantly less than that observed in Bluetooth transmission protocols;
(3) High stability, second-level data transmission, no packet loss, and self-repair capability even if data are lost.

These features are intrinsically linked to the wireless transmission principle used in this smart toolholder, specifically the lossless extended range synchronized (LXRS) and LXRS+ communication protocols. These protocols enable users to achieve near-lossless data acquisition in most environments using data buffering, wireless acknowledgment, and retransmission mechanisms. Each node stores the collected data along with timestamps in an internal 2 Mbit FIFO buffer, from which the data are subsequently retrieved for transmission. Upon receipt of a data packet, the gateway issues an acknowledgment; the node retransmits the data until the acknowledgment is confirmed. The inherent overhead of the transmission scheduling protocol ensures that the node has sufficient recovery time when the quality of wireless communication is poor. This capability facilitates lossless performance even in environments where node packet error rates approach 50% and supports operation when the gateway and nodes intermittently move in and out of each other’s coverage areas. It is important to note that lossless functionality is contingent upon synchronization being enabled; if the application demands stable latency or can tolerate data loss, the lossless feature may be disabled.

**Figure 2:**
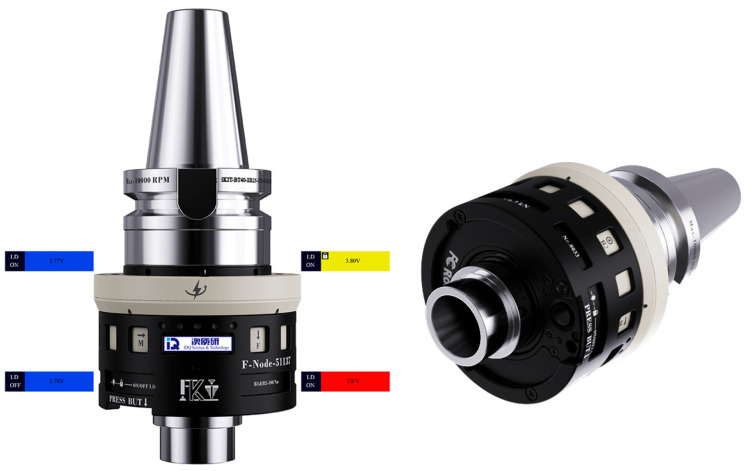
Monitoring the screen display and the actual bottom appearance of wireless smart toolholder.

Signal transmission relies on the Lossless Extended Range Synchronized (LXRS) communication protocol in this study. This protocol utilizes data buffering and wireless acknowledgment mechanisms. In typical industrial environments, lossless transmission is guaranteed under conditions of up to 15,000 rpm rotational speed, within a 5-m line-of-sight distance, and under standard electromagnetic interference. If the application demands ultra-low stable latency and the lossless synchronization feature is disabled, the actual packet error rate remains strictly controlled below 2%. The system supports an adjustable sampling frequency of up to 2048 Hz. While ultra-high-frequency regenerative chatter may require higher rates, a sampling frequency of 2048 Hz yields approximately 11.7 measurement points per revolution at a spindle speed of 10,500 rpm. This resolution is highly sufficient for extracting macroscopic process trends, monitoring tooth-passing frequencies, and conducting in-depth analyses of load variations. Other features include:

(1) The transmission can achieve high-frequency and high-precision monitoring with a sampling frequency range of 8–2048 Hz, which is adjustable in the software;
(2) No crosstalk between the signals, which can be controlled to within 5%, ensuring the accuracy and reference value of the acquired data;
(3) A display screen to present dynamic information (Figure 2): when operated in combination with buttons, the screen can exhibit corresponding effects such as startup animation and battery status indicators (blue signifies normal, whereas red and yellow denote low battery for the handle and charging conditions, respectively).

**Figure 3:**
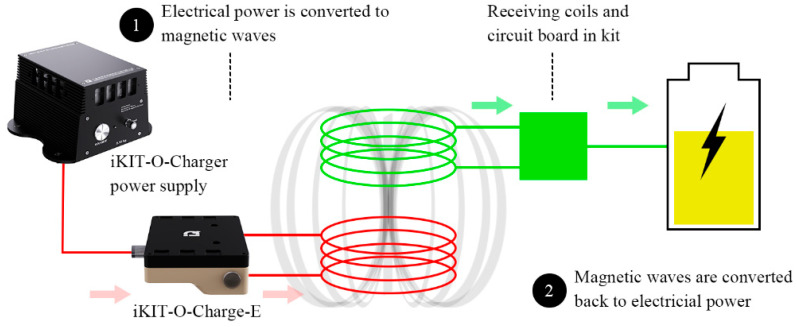
Wireless power supply schematic diagram.

To ensure continuous data acquisition without depleting battery life during long manufacturing cycles, a non-contact inductive charging module is integrated (Figure 2 and Figure 3). The resulting 5V direct current is supplied to the battery, allowing uninterrupted high-frequency data transmission even under harsh, wet cutting conditions. The power-supply mechanism is illustrated in Figure 3. The transmitter component comprises an induction module charger (iKIT-O-Charge-E) and an iKIT-O-Charger power supply unit. Upon activation, the power source is converted into a high-frequency current by the transmitting circuit. This high-frequency alternating current passes through the transmitting coil, generating an oscillating magnetic field. The receiver, integrated within the toolholder, contains a power management chip, a power amplifier chip, and an electromagnetic coil. The oscillating magnetic field induces an alternating current in the receiving coil, which is then rectified into a direct current by the receiving circuitry. The resulting 5 V direct current is supplied directly to the battery, thereby ensuring sustained power delivery. When the toolholder enters charging mode, the display screen is illuminated in yellow (Figure 3). During machining operations, the toolholder’s continuous rotation produces a flashing yellow light strip effect that contrasts with the color scheme of the main body, highlighting a distinctive and informal aesthetic while maintaining functional priorities. In addition, all receiver components are encapsulated with an adhesive and the transmission cables are waterproof, thereby guaranteeing reliable operation in harsh environments or conditions involving exposure to cutting fluids.

### 2.2. Testing Method

A comprehensive cutting experimental platform is established using a high-precision three-axis CNC vertical milling machine (Mazak). The workpiece material is 6061-T6 aluminum alloy (dimensions: 80 mm × 80 mm × 40 mm). A commercially available solid-carbide three-flute flat-end mill with a diameter of 4 mm (Meihua, China) was used. The radial runout of the clamped tool was calibrated to a maximum of 0.008 mm, representing a realistic and acceptable maximum tolerance for industrial roughing and semi-finishing operations to avoid inducing excessive artificial vibrations. All cutting tests are conducted under standard emulsion cutting fluid conditions, with a continuous flow rate of 10 L/min at a pressure of 2 bar directed at the cutting zone, ensuring that the toolholder’s waterproof sealing was rigorously tested.

This study examines the effects of different milling process parameters, such as spindle speed (*n*), feed rate (*f*), and cut depth (*a_p_*), on the characteristics of data obtained from the intelligent toolholder (axial force, torque, and two-way bending moments. The cutting operation is performed using a reciprocating-side milling approach. To ensure the reliability and consistency of the experimental data, specific continuous machining paths are programmed into the CNC controller using G-code. A single-factor variable method combined with a gradient testing strategy is used, resulting in eight distinct combinations of process parameters, as listed in Table 1. Throughout the cutting trials, the radial cutting width (*a_e_*) or length of a single pass is maintained at 15 mm. A cutting length of 15 mm per pass is specifically selected; this distance is sufficient to achieve stable, steady-state mechanical signal output while preventing excessive bulk thermal buildup in the workpiece, which could obscure the purely mechanical effects of the parameter variations. For each parameter set, the machine tool continuously performed one complete cycle of reciprocating cuts to acquire a sufficiently long steady-state cutting signal and minimize random errors. To ensure statistical reliability and demonstrate data reproducibility, each parameter set involved four repeated reciprocating cutting passes. The data presented in the subsequent sections represent the average values extracted from the steady-state regions of these repeated trials.

In this study the fundamental machine input parameters are controlled as well as the computed cutting linear velocity ($v_{c}$), feed per tooth ($f_{z}$), and material removal rate (MRR), to further clarify the mechanisms by which variations in cutting conditions influence the monitoring signal characteristics [19]. These parameters are critical because they directly affect the stress state within the cutting deformation zone, generation of cutting heat, and the mechanical load imposed on the tool. The cutting linear velocity represents the instantaneous linear speed of the point on the main cutting edge of the milling cutter that is farthest from the axis of rotation relative to the workpiece surface. This parameter is a pivotal factor influencing the cutting temperature and tool wear rate [20]. Generally, higher cutting speeds tend to increase cutting forces and temperatures, thereby accelerating tool wear. The cutting linear velocity is calculated using $v_{c}=\frac{\pi \cdot D\cdot n}{1000}$ [21], where *D* is the diameter of the milling cutter, which is 4 mm in this experiment.

Feed per tooth is defined as the linear displacement of the workpiece relative to the milling cutter along the feed direction during the angular distance of cutter rotation corresponding to one tooth. This parameter is a fundamental geometric factor that influences both the thickness of the undeformed chip and the cutting force exerted by a single cutting edge. This can be determined using $f_{z}=\frac{f}{n\cdot z}$ [22], where *z* is the effective number of cutting edges of the milling cutter (*z* = 3 in the present study). Previous research has demonstrated a significant positive correlation between the feed per tooth and the cutting force. An increase in the feed per tooth results in a proportional increase in the cross-sectional area of the cutting layer, thereby augmenting the resistance to lattice shear deformation and frictional forces that the cutting edge must overcome during material removal [23]. Despite the absolute cutting force increasing with the feed per tooth, the specific cutting energy, defined as the energy required to remove a unit volume of material, decreases as the feed per tooth increases. This phenomenon occurs because at very low values of the feed per tooth, the plowing effect induced by the cutting-edge radius predominates, causing the material to experience extrusion friction rather than pure shear deformation. Consequently, when the feed per tooth surpasses a critical threshold, the cutting mechanism transitions to a regime dominated by pure shear, thereby enhancing cutting efficiency. The material removal rate (MRR, $MRR=a_{p}\cdot a_{e}\cdot f$) refers to the volume of material removed from the workpiece per unit time. It is an important macroscopic indicator for measuring the cutting efficiency and evaluating the overall cutting power consumption of a machine tool. Detailed process parameters and calculation results for the above cutting parameters are listed in Table 1.

## 3. Result and Discussion 

### 3.1. Rotational Speed Effect

**Figure 4:**
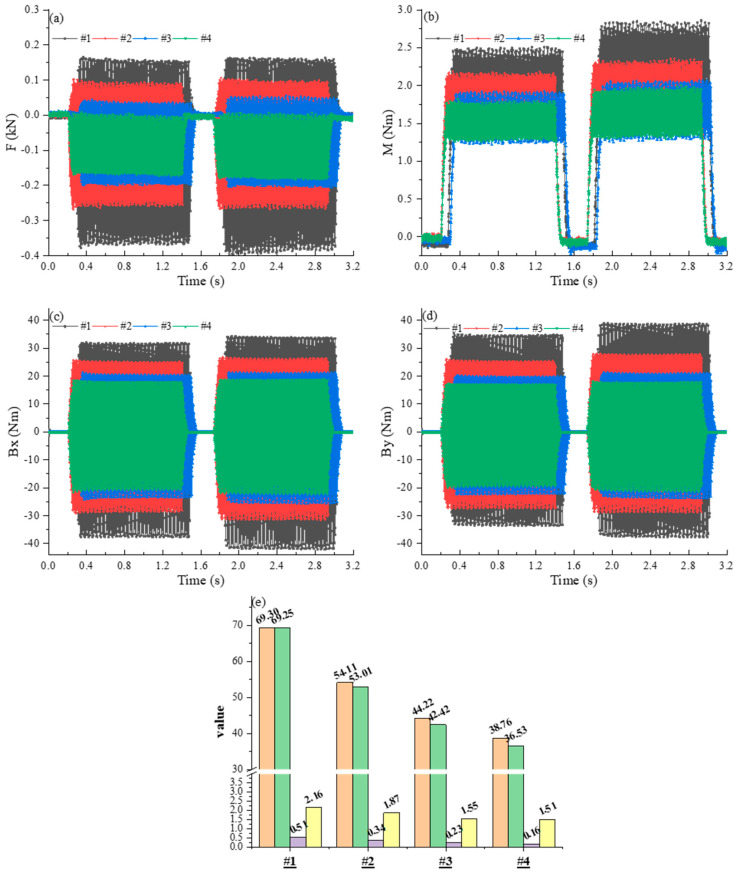
In situ monitoring data under different *n*: (**a**) *F*; (**b**) *M*; (**c**) *B_x_*; (**d**) *B_y_*; (**e**) Consolidated bar charts at four different speeds (see Table 1).

Figure 4 displays the real-time data collected across varying spindle speeds, reflecting different cutting speeds ($v_{c}$). To address potential random errors, standard deviations from the four repeated cutting trials are calculated, and corresponding error bars are incorporated into the consolidated bar charts (e.g., Figure 4e), demonstrating excellent data reproducibility. A previous study [14] showed that this intelligent toolholder exhibits no signal crosstalk under static calibration conditions, which is achieved through structural enhancements and optimizations. Consequently, the data analyses reported herein are deemed reliable.

**Figure 5:**
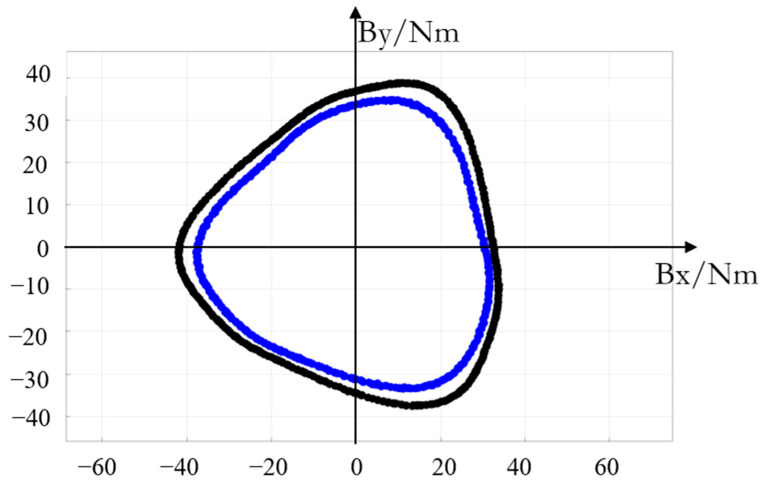
Polar plots; black contour and blue contour correspond to the conventional and climb milling process.

The reciprocating motion consisted of alternating climb milling and conventional milling passes. It was systematically observed that all monitored parameters significantly increased during the conventional milling phase. The host software visualizes the biaxial bending moments as a planar spatial trajectory (Figure 5). This Lissajous curve representation maps $B_{x}$ against $B_{y}$, providing a qualitative assessment of the resultant radial force vector. The black contour area (conventional milling) is substantially larger than the blue contour (climb milling). In conventional milling, the cutter teeth initiate contact at zero chip thickness, resulting in prolonged plowing and severe frictional rubbing before pure shear material removal begins. This intensifies the radial repulsive force acting on the tool overhang, directly leading to the elevated bending moments captured by the smart toolholder. This finding demonstrates that the intelligent toolholder can accurately capture macroscopic variations in the microscopic contact mechanics of cutting.

The increase in the bending moment signals is partially attributable to the substantial frictional resistance and radial repulsion generated by the extended plowing process during the initiation phase of conventional milling. This necessitates additional energy input from the cutting system to overcome the frictional work, resulting in a marked increase in the bidirectional bending moment [24]. Moreover, the bidirectional bending moment measured by the device is predominantly determined by the product of the radial cutting force and overhang length of the tool. In conventional milling, the cutter teeth move opposite to the workpiece feed direction, whereas in climb milling, the cutting force vector tends to press the workpiece against the worktable, thereby facilitating a smoother cutting process. The significantly increased radial repulsive force during conventional milling acts directly on the overhang of the milling cutter, causing a substantial increase in *B_x_* and *B_y_*, which are transmitted to the base of the intelligent toolholder. This phenomenon explains why conventional milling is prone to induce minor bending deformations and high-frequency chatter within the toolholder.

When examining the influence of rotational speed, all four measured signals decrease as the rotational speed gradient increases (Figure 4). To facilitate a detailed analysis of the effects of process parameters on the monitoring data, we isolated the data corresponding to the climb milling phase and computed the average peak-to-peak value of the bending moment. As illustrated in Figure 4e, when the rotational speed was elevated from 6000 rpm to 10,500 rpm, the average peak-to-peak values of *B_x_* decreased from 69.3 Nm to 38.76 Nm, the average peak-to-peak value of *B_y_* declined from 69.25 Nm to 36.53 Nm, the average M value reduced from 2.16 Nm to 1.51 Nm, and the average peak-to-peak value of F diminished from 0.51 kN to 0.16 kN. The parameter matrix (188 to 330 m/min) was strategically selected because it covers the critical transition zone for aluminum alloys. At lower speeds, the strong adhesion characteristics of 6061-T6 aluminum promote the formation of built-up edges (BUE) on the tool’s rake face [25], which increases the effective friction coefficient and causes higher load fluctuations. As the cutting speed increases, the accelerated plastic deformation per unit time elevates the temperature within the primary shear zone. This induces the thermal softening of the aluminum matrix, lowering its yield strength [26]. Consequently, the material requires less tangential shearing energy to be removed, which macroscopically manifests as the distinct reduction in torque and bending moments successfully captured by the toolholder.

### 3.2. Feed Rate Effect

**Figure 6:**
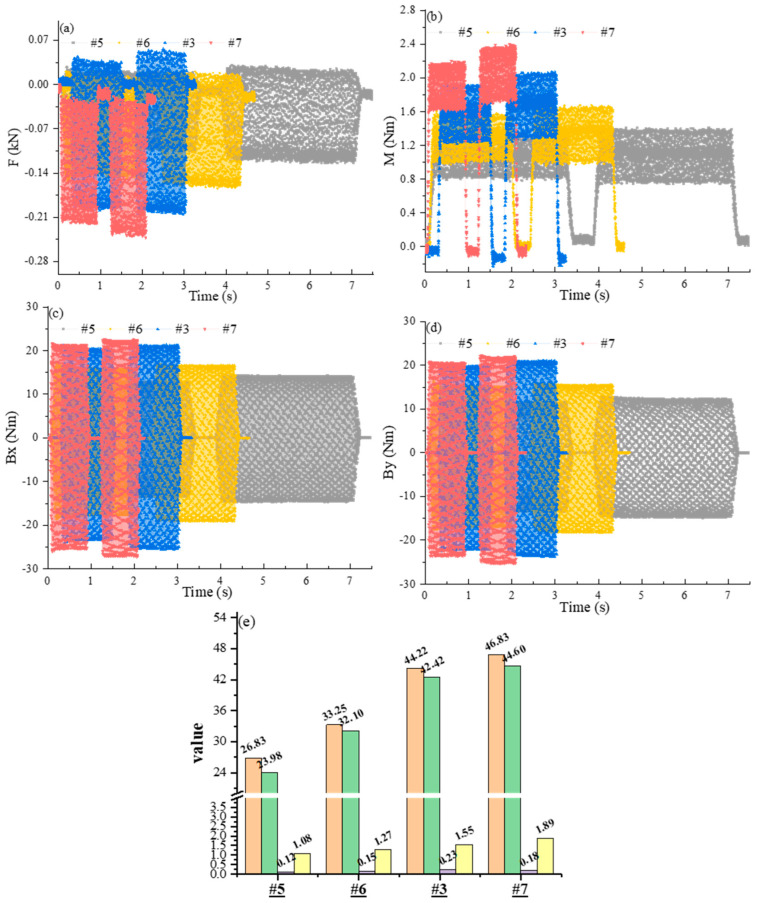
In situ monitoring data under different *f*: (**a**) *F*; (**b**) *M*; (**c**) *B_x;_* (**d**) *B_y_*; (**e**) Consolidated bar charts at four different feed rates (see Table 1).

At a constant cutting speed, increasing the feed rate linearly augments the maximum undeformed chip thickness ($h_{ex}$), which is directly proportional to the feed per tooth ($f_{z}$). As detailed in Table 1, increasing the feed rate from 1500 mm/min to 5500 mm/min corresponds to a multi-fold increase in $f_{z}$. Specifically, when the feed rate increased from 1500 mm/min to 5000 mm/min, the feed per tooth correspondingly increased from 0.0556 mm/tooth to 0.2037 mm/tooth, representing a more than a three-fold increase escalation. Figure 6 illustrates that, despite a substantial increase in feed rate, the peak value of the cutting force exhibits only a modest change. In contrast, the average torque demonstrates a more pronounced increase, indicating a positive correlation between feed rate and torque. An elevated feed rate directly enhances the material removal rate per unit time. To effectively remove thicker sections of the aluminum alloy, the cutting tool must exert a greater tangential shearing force to overcome the lattice bonding forces of the material. Consequently, the monitoring toolholder detects a significant increase in the torque signals as the feed rate increases.

This behavior is fundamentally linked to the material removal mechanics. An elevated feed per tooth directly increases the cross-sectional area of the shear layer [27]. To effectively overcome the lattice bonding forces of the thicker aluminum section, a greater tangential cutting force is required, which is precisely reflected in the near-linear increase in M. Furthermore, a higher feed rate increases the volume of material compressed normal to the cutting edge. The enhanced elastic recovery of the workpiece exerts a stronger radial resistance against the tool. When coupled with the tool’s overhang length, this dramatically amplifies the lateral bending moments. As shown in Figure 6e, with standard deviations confirming the trend’s stability, the average peak-to-peak value of $B_{x}$ increased from 26.83 Nm to 46.83 Nm. This confirms the system’s sensitivity in decoupling the plowing effect from pure shear deformation as chip thickness varies.

### 3.3. Cut Depth Effect

**Figure 7:**
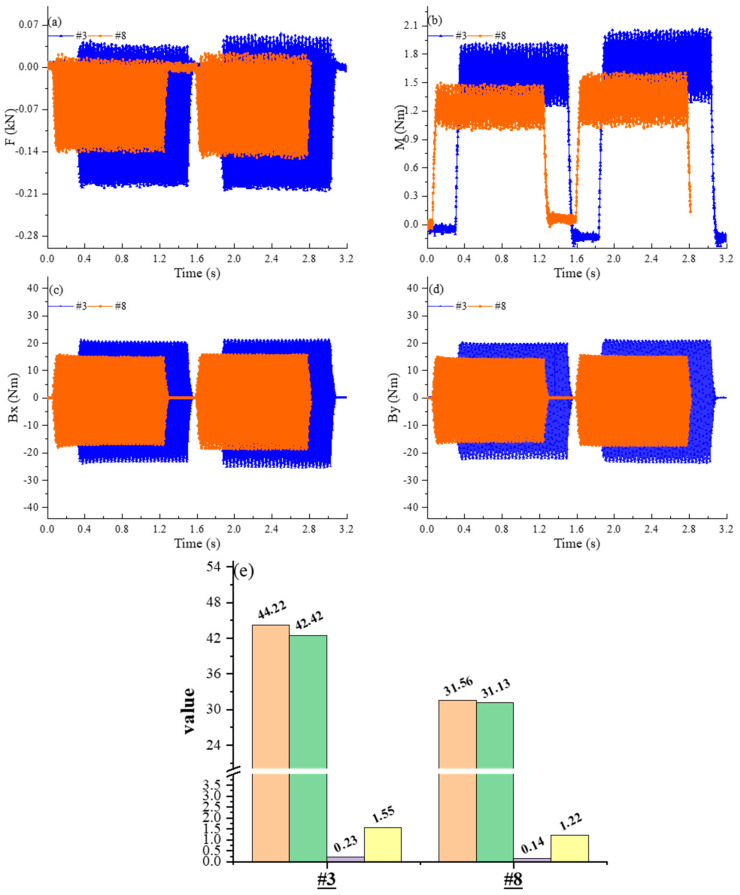
In situ monitoring data under different *a_p_*: (**a**) *F*; (**b**) *M*; (**c**) *B_x_*; (**d**) *B_y_*; (**e**) Consolidated bar charts at two different depths of cut (see Table 1).

Figure 7 illustrates the multidimensional signals monitored under varying $a_{p}$. With the cutting speed and feed rate held constant, reducing $a_{p}$ from 1.5 mm to 1.0 mm proportionally decreases the active length of the helical cutting edge engaged with the workpiece. Consequently, the total tangential resistance required to sever the material decreases linearly, which is perfectly mirrored by the reduction in the monitored M shown in Figure 7e. A reduction in the depth of cut leads to a proportional decrease in the overall resultant cutting force along both the feed and radial directions. Given that the variation in the lever arm length, which is defined as the distance from the toolholder base to the cutting zone, is minimal, the bending moment transmitted to the strain-sensitive region of the extrasensory toolholder diminishes. 

Interestingly, the biaxial bending moments exhibit a complex response to depth variations, which highlights the advantage of multi-axis monitoring. Greater depths of cut significantly alter the tool’s dynamic stiffness response. Due to the high specific strength of the aluminum alloy, a larger engagement length exacerbates dynamic interactions between the tool flank and the machined surface. This can induce micro-regenerative chatter, causing the alternating current (AC) component of the bending moment signals to exhibit wider fluctuation bands. The smart toolholder’s ability to capture these spatial load asymmetries indicates its potential for real-time chatter prediction.

## 4. Conclusions

This study systematically investigated the causal relationships between milling process parameters and multidimensional mechanical responses using an advanced wireless smart toolholder. By moving beyond conventional single-axis measurements, the in-depth parametric analysis of 6061-T6 aluminum alloy machining yields the following principal conclusions:

(1) The non-contact inductive power supply and structurally balanced design (ISO 1940-1 G2.5) effectively resolve the operational bottlenecks of continuous multidimensional telemetry in high-speed, fluid-intensive enclosed CNC environments. The hardware ensures high-fidelity, synchronous acquisition of axial force, torque, and biaxial bending moments with high statistical reproducibility.
(2) The system accurately quantified the transition of material removal mechanisms. Torque and biaxial bending moments exhibit a strong positive correlation with feed per tooth and axial depth of cut, dictated by the increased shear area and radial elastic recovery. Conversely, these mechanical loads are negatively correlated with cutting speed, sensitively capturing the macroscopic load reduction driven by the thermal softening of the aluminum matrix and the suppression of built-up edges.
(3) The independent decoupling and planar visualization (Lissajous trajectories) of biaxial bending moments reveal the spatial load asymmetries between conventional and climb milling phases. This capability confirms that the proposed multidimensional monitoring framework transcends traditional load measurement, providing a robust, highly sensitive data foundation for future intelligent condition monitoring, tool wear prediction, and adaptive chatter control.

While the proposed wireless smart toolholder demonstrates significant advantages for in situ monitoring, it is essential to acknowledge alternative sensing technologies. Traditional stationary table dynamometers offer ultra-high bandwidth and extreme precision but are strictly limited by workpiece dimensions and cannot be applied in rotating operations. Conversely, indirect monitoring via spindle currents is highly non-intrusive but lacks the micro-mechanical sensitivity required to detect minor load variations at the cutting zone. Despite successfully bridging these gaps, the current toolholder design presents certain limitations. The maximum sampling frequency of 2048 Hz, while adequate for macroscopic load trends and tooth-passing frequencies, may restrict the capture of ultra-high-frequency regenerative chatter in precision micro-milling. Additionally, although inductive charging effectively extends operational time, complete energy autonomy is not yet realized. Future work will focus on integrating kinetic energy harvesting mechanisms (e.g., piezoelectric or electromagnetic harvesters) to achieve a self-sustained system. Furthermore, deploying edge computing algorithms directly within the toolholder’s built-in microprocessor is planned, which will enable real-time, AI-driven tool wear diagnostics and adaptive control without heavily relying on continuous high-bandwidth wireless transmission.

## Figures and Tables

**Table 1:** Milling process parameters.

	$v_{c}$ m/min	$f_{z}$ mm/z	*f* mm/min	*n* rpm	*a_p_* mm	MRR cm^3^/min
#1	188.4	0.2222	4000	6000	1.5	90
#2	235.5	0.1778	4000	7500	1.5	90
#3	282.6	0.1481	4000	9000	1.5	90
#4	329.7	0.1270	4000	10,500	1.5	90
#5	282.6	0.0556	1500	9000	1.5	33.75
#6	282.6	0.0926	2500	9000	1.5	56.25
#7	282.6	0.2037	5500	9000	1.5	123.75
#8	282.6	0.1481	4000	9000	1	60

## Data Availability

The data presented in this study are available on request from the corresponding author.
